# High-Performance Aqueous Zinc–Manganese Battery with Reversible Mn^2+^/Mn^4+^ Double Redox Achieved by Carbon Coated MnO_*x*_ Nanoparticles

**DOI:** 10.1007/s40820-020-00445-x

**Published:** 2020-05-13

**Authors:** Jingdong Huang, Jing Zeng, Kunjie Zhu, Ruizhi Zhang, Jun Liu

**Affiliations:** 1 School of Materials Science and Engineering, Central South University, Changsha, 410083 People’s Republic of China; 2 Key Laboratory of Advanced Energy Materials Chemistry (Ministry of Education), College of Chemistry, Nankai University, Tianjin, 300071 People’s Republic of China; 3 Hunan Institute of Technology, Hengyang, 421002 People’s Republic of China

**Keywords:** Aqueous zinc–manganese batteries, Mn-based cathode materials, High energy density, Mn^2+^/Mn^4+^ double redox

## Abstract

There is an urgent need for low-cost, high-energy-density, environmentally friendly energy storage devices to fulfill the rapidly increasing need for electrical energy storage. Multi-electron redox is considerably crucial for the development of high-energy-density cathodes. Here we present high-performance aqueous zinc–manganese batteries with reversible Mn^2+^/Mn^4+^ double redox. The active Mn^4+^ is generated in situ from the Mn^2+^-containing MnO_*x*_ nanoparticles and electrolyte. Benefitting from the low crystallinity of the birnessite-type MnO_2_ as well as the electrolyte with Mn^2+^ additive, the MnO_*x*_ cathode achieves an ultrahigh energy density with a peak of 845.1 Wh kg^−1^ and an ultralong lifespan of 1500 cycles. The combination of electrochemical measurements and material characterization reveals the reversible Mn^2+^/Mn^4+^ double redox (birnessite-type MnO_2 _↔ monoclinic MnOOH and spinel ZnMn_2_O_4_ ↔ Mn^2+^ ions). The reversible Mn^2+^/Mn^4+^ double redox electrode reaction mechanism offers new opportunities for the design of low-cost, high-energy-density cathodes for advanced rechargeable aqueous batteries.

**Electronic supplementary material:**

The online version of this article (10.1007/s40820-020-00445-x) contains supplementary material, which is available to authorized users.

## Highlights

- Aqueous zinc-manganese batteries with reversible Mn^2+^/Mn^4+^ double redox are achieved by carbon-coated MnO_*x*_ nanoparticles.
- Combined with Mn^2+^-containing electrolyte, the MnO_*x*_ cathode achieves an ultrahigh energy density with a peak of 845.1 Wh kg^−1^ and an ultralong lifespan of 1500 cycles.
- The electrode behaviors and reaction mechanism are systematically discussed by combining electrochemical measurements and material characterization.

**Electronic supplementary material:**

The online version of this article (10.1007/s40820-020-00445-x) contains supplementary material, which is available to authorized users.

## Graphical abstract

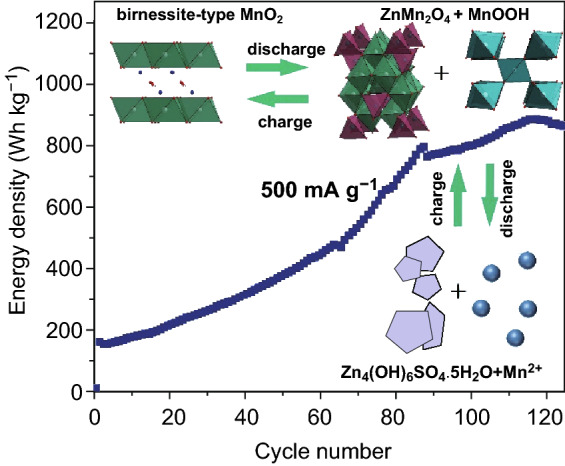

## Introduction

Considering the projected climatic deterioration, pollution, and inherent limit of fossil fuels, focus toward more environmentally friendly and sustainable energy sources continues to grow [1, 2]. Nevertheless, the utilization of sustainable energy sources such as solar, water, and wind requires a safe, efficient, and economic energy conversion system that can smoothen the intermittency of sustainable energy [3]. Although current lithium-ion batteries (LIBs) have dominated the portable energy market, their large-scale grid application is limited by the high cost and scarcity of Li resources and safety concerns associated with flammable organic electrolytes that lead to thermal runaway [4–6]. Recently, rechargeable aqueous zinc-based batteries have been considered candidates for stationary grid-level storage of the intermittent renewable energies due to their low cost, improved safety, simpler manufacturing conditions, and greener operation [7, 8].

As for the low cost, non-toxicity, and high theoretical capacity, Mn-based materials are considered as ideal cathode materials for aqueous zinc-ion batteries (AZIBs) [9, 10]. Current studies focus on crystallographic tunnel-type structures MnO_2_, including α-MnO_2_, β-MnO_2_, γ-MnO_2_, and other types [11–16]. Additionally, spinel-type Mn_3_O_4_ and ZnMn_2_O_4_ show as viable cathode materials for AZIBs [17–20]. Recently, due to its larger capacity and higher metal ion diffusion rate, layered MnO_2_ is considered to be a more promising cathode material [21]. However, most of the MnO_2_ that has been reported only utilizes the electron during Mn^4+^/Mn^3+^ conversion, therefore those cathode materials fall short of meeting the demands for portable and large-scale stationary energy storage systems. The Mn^2+^/Mn^4+^ double redox is observed in the tunnel-type γ-MnO_2_ [22]. During the discharge process, spinel-type ZnMn_2_O_4_, tunnel-type γ-Zn_*x*_Mn^2+^O_2_, and layered-type L-Zn_*y*_Mn^2+^O_2_ are generated in sequence, and a high capacity of 285 mAh g^−1^ can be achieved. The structural variation is reversible, but the tunnel-type γ-MnO_2_ suffers from poor electrical and ionic conductivities [23]. Therefore, it is still highly infusive to discover potential satisfactory Mn-based cathode materials for energy storage.

**Fig. 1:**
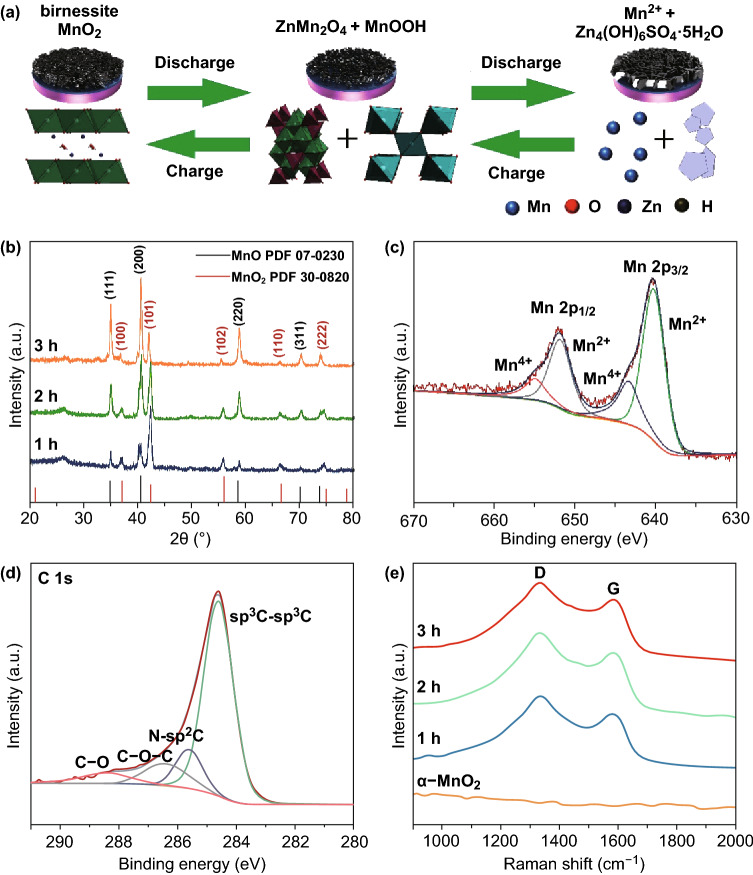
**a** Working principle of Zn/MnO_*x*_ battery. **b** XRD patterns of MnO_*x*_. XPS spectra of MnO_*x*_-2: **c** high resolution of Mn 2*p* and **d** high resolution of C 1*s*. **e** Raman spectra of the MnO_*x*_ and α-MnO_2_

Herein, we propose the use of carbon-coated MnO_*x*_ nanoparticles as a cathode material for zinc–manganese batteries. In these batteries, the active low-crystallinity birnessite-type MnO_2_ is generated in situ from the Mn^2+^-containing MnO_*x*_ nanoparticles and electrolyte during the charge process. Owing to the lower crystallinity, the active birnessite-type MnO_2_ contains higher energy and possesses the ability to achieve Mn^2+^/Mn^4+^ double redox [24]. In addition, the small particle size of MnO_*x*_ and the high conductivity of the carbon substrates provide good conditions for the oxidation reactions. Benefitting from the Mn^2+^/Mn^4+^ double redox, the MnO_*x*_ cathode using Mn^2+^-containing ZnSO_4_ electrolyte exhibits an ultrahigh energy density with a peak of 845.1 Wh kg^−1^ and an ultralong lifespan of 1500 cycles. A detailed investigation is also performed to analyze the mechanism of the reversible Mn^2+^/Mn^4+^ double redox. This working principle of the zinc–manganese battery is illustrated in Fig. 1a. These findings may offer new opportunities to design low-cost and high-performance aqueous zinc–manganese batteries for large-scale energy storage.

## Experimental Section

### Synthesis of α-MnO_2_

The α-MnhO_2_ was synthesized using a hydrothermal procedure [25]. Firstly, KMnO_4_ (0.7 g) was dissolved in deionized water (70 mL); then, concentrated HCl (3.3 mL) was added into the solution under continuous vigorous stirring at room temperature for 10 min. The final solution was transferred into a Teflon-lined stainless-steel autoclave (100 mL) and maintained at 140 °C for 16 h. Next, the brown product was collected by centrifugation and washed with deionized water and ethanol for three times. Finally, the brown product was dried at 70 °C for 24 h.

### Synthesis of MnO_*x*_ and MnO

In a typical procedure, α-MnO_2_ nanorods (0.04 g) were dispersed in ethanol (10 mL) with 2-methylimidazole (2 g) dissolved. The obtained suspension was dried in a drying oven at 80 °C for 24 h. Then the dried sample was carefully ground by agate mortar. After that, the powders were heated at 700 °C for 1, 2, or 3 h at a rate of 2 °C min^−1^ in a tube furnace under a flowing Ar atmosphere to obtain MnO_*x*_-1, MnO_*x*_-2, or MnO_*x*_-3. Besides, the MnO was obtained by heating the brown powders at 700 °C for 2 h at a rate of 2 °C min^−1^ in a tube furnace under a flowing Ar/H_2_ atmosphere.

### Materials Characterization

X-ray diffraction (XRD) measurements were performed on a Rigaku D/max 2500 powder diffractometer with monochromatic Cu-Kα radiation and the wavelength of 1.54178 Å. SEM and transmission electron microscope (TEM) images were taken using a FEI Helios Nanolab G3 UC and TEM JEOLJEM-2100 electron microscope, respectively. The elementary composition and valence state of samples were characterized by X-ray photoelectron spectroscope (XPS, Thermo ESCALAB 250Xi, monochromatic Al-Kα radiation). Raman spectra were collected on an Invia Raman spectrometer, using an excitation laser of 514.5 nm. ICP-OES spectrometer (SPECTRO BLUE SOP) was carried out to determine the concentration of Mn and S elements.

### Electrochemical Measurements

The electrochemical measurements were tested by assembly of CR2032-type coin cells in air atmosphere. The working electrode film was prepared by coating the slurry on a Ti foil, and the slurry consisted with active materials, polyvinylidene fluoride (PVDF) binder, super P additive (7: 2: 1). The mass loading of active materials is around 1.5 mg cm^−2^. Zn foil was used as the counter electrode. 1 M ZnSO_4_ and 0.3 M MnSO_4_ solution were used as electrolyte. Cyclic voltammetry (CV) curves were recorded on an electrochemical workstation (CHI660E). The galvanostatic discharge–charge tests were performed on a Land CT 2001A tester in a potential window of 0.8–1.8 V.

## Results and Discussion

### Structural Characterization

The crystallographic structure and the phase composition of the pre-reduced MnO_*x*_ are examined by XRD measurement. As shown in Fig. 1b, the diffraction peaks of manganese oxides indicate a crystalline hybrid, which match well with simulated MnO_2_ (JCPDS Card No. 30-0820) and MnO (JCPDS Card No. 07-0230). The XRD results clearly show that the ratios of MnO to MnO_2_ in the products calcined at different reaction time are completely different. The synthesized manganese oxides are labeled MnO_*x*_-1, MnO_*x*_-2, and MnO_*x*_-3, respectively. The XRD analysis of the α-MnO_2_ and MnO is also shown in Fig. S1a, b.

In order to further analyze the manganese valence states of MnO_*x*_ and α-MnO_2_, the samples were analyzed by X-ray photoelectron spectroscopy (XPS) (Figs. 1c, d, S2). The high-resolution XPS spectrum of Mn 2*p* for MnO_*x*_ composite displays four peaks with binding energies at 640.35 eV (651.92 eV) and 643.50 eV (654.92 eV), which correspond to Mn^2+^ and Mn^4+^, respectively [26]. This result further proves that the pre-reduced MnO_*x*_ is a composite of MnO_2_ and MnO. For MnO_*x*_-1, MnO_*x*_-2, and MnO_*x*_-3, the fractions of Mn^2+^ are ≈ 64.1%, 71.4%, and 79.3%, respectively. As shown in Fig. 1d, the high-resolution XPS spectrum of C 1 *s* for MnO_*x*_ composite can be fitted into four parts, including the peaks located at 288.4, 286.5, 285.5, and 284.5 eV, corresponding to C–O, C–O–C, N–*sp*^2^C, and *sp*^3^C–*sp*^3^C bonds, respectively [27]. The Raman spectrum is given in Fig. 1e. The broad peaks located at 1332 and 1586 cm^−1^ are related to the D band and G band of carbon, respectively. The high intensity of the D band indicates the presence of defects and non-graphitic carbon in the carbon coating [28].

**Fig. 2:**
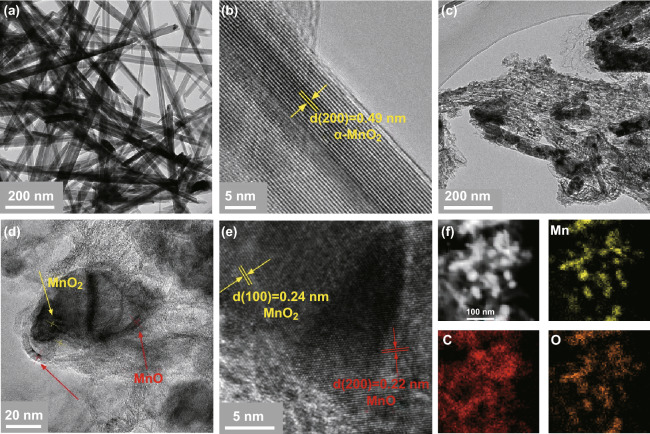
**a** TEM and **b** HRTEM images of α-MnO_2_. **c** TEM image, **d, e** HRTEM images, and **f** EDX elemental mapping images of MnO_*x*_-2

The morphology of as-prepared α-MnO_2_ precursors is assessed by TEM, showing a nanorod shape for α-MnO_2_ (Fig. 2a). The high-resolution (HR) TEM image (Fig. 2b) possesses regular lattice fringes with *d*-spacing of 0.49 nm, corresponding to the interplanar distance of (200) plane of α-MnO_2_. After the composite powder is calcined, the morphology of α-MnO_2_ changes to smaller nanoparticles coated with carbon (Fig. 2c). The MnO_*x*_ nanoparticles are highly dispersed in the carbon substrate and form better contact with the electrolyte, thereby establishing a highly conductive network for the electrons and further providing good conditions for the oxidation reaction of MnO_*x*_ and Mn^2+^ ions [29]. HRTEM images (Fig. 2d, e) reveal that MnO_*x*_ possesses regular lattice fringes spacing of 0.24 and 0.22 nm, corresponding to (100) plane of MnO_2_, and (200) plane of MnO, respectively. The high-angle annular dark-field (HAADF)-STEM image and energy-dispersive X-ray (EDX) elemental mapping images (Fig. 2f) of MnO_*x*_ confirm the dispersion of small MnO_*x*_ nanoparticles in the carbon coating.

### Electrochemical Characterization

**Fig. 3:**
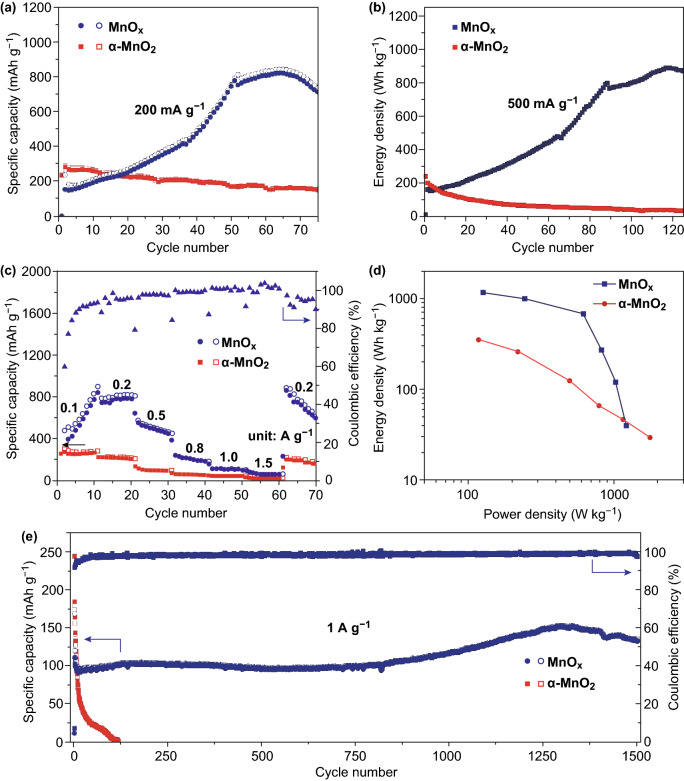
Cycling performance of MnO_*x*_-2 and α-MnO_2_ **a** at 0.2 A g^−1^ and **b** at 0.5 A g^−1^. **c** Rate performance of MnO_*x*_-2 and α-MnO_2_. **d** Ragone plot and **e** long cycling performances at 1.0 A g^−1^ of MnO_*x*_-2 and α-MnO_2_ cathode

Figure 3a compares cycling performance between MnO_*x*_-2 and α-MnO_2_ cathodes at 0.2 A g^−1^. Drastic capacity fade can be clearly seen in the curves of α-MnO_2_, maintaining 154.5 mAh g^−1^ after 75 cycles. With respect to MnO_*x*_-2 electrode, the initial charge capacity is 156.3 mAh g^−1^ due to the electrochemical oxidation of Mn^2+^. After 75 cycles, the MnO_*x*_-2 electrode achieves specific capacity up to 714.7 mAh g^−1^ (based on the active material initial mass of cathode). The capacity of MnO_*x*_-2 exceeding its theoretical capacity can be attributed to the addition of Mn^2+^ in the electrolyte. The Mn^2+^ added in the electrolyte can also participate in the reversible Mn^2+^/Mn^4+^ double redox, so the capacity of MnO_*x*_-2 tops its theoretical capacity. In addition, MnO_*x*_-2 cathode displays a gradually increasing of specific capacity, possibly due to the following reason: The MnO in the MnO_*x*_ is gradually oxidized during each charging process. And the newly formed MnO_2_ can also achieve reversible Mn^2+^/Mn^4+^ double redox to increase the specific capacity. This phenomenon is commonly observed in transition metal oxides [30, 31]. The voltage profiles of MnO_*x*_-2 are shown in Fig. S6. As shown in Fig. S6, the voltage profiles of this electrode do not change significantly in the first 50 cycles. During the capacity decay, however, there are some changes in the voltage profiles of the electrode, which may be due to changes of electrode materials.

As shown in Fig. 3b, the MnO_*x*_-2 electrode using Mn^2+^-containing electrolyte exhibits an ultrahigh energy density with a peak of 845.1 Wh kg^−1^ at 500 mA g^−1^. Furthermore, the rate capabilities are compared at increased current densities (Fig. 3c). The MnO_*x*_ electrode exhibits capacities of 844.5 mAh g^−1^ at 0.1 A g^−1^ after 10 cycles. As currents increase from 0.1 to 1.5 A g^−1^, for MnO_*x*_ electrode, capacities of 844.5, 783.6, 551.1, 226.8, 114.8, and 59.7 mAh g^−1^ are delivered. For comparison, the α-MnO_2_ electrode fades drastically from 270.7 (0.1 A g^−1^) to 27.2 mAh g^−1^ (1.5 A g^−1^). Upon rate recovery to 0.2 A g^−1^, a reversible capacity of 863 mAh g^−1^ is restored for MnO_*x*_ electrode. Moreover, the MnO_*x*_ electrode displays higher energy density (1158 Wh kg^−1^) and power density (1212 W kg^−1^) in the Ragone plot in comparison with α-MnO_2_ cathode for aqueous ZIBs as shown in Fig. 3d. When the MnO_*x*_ is cycled 1500 times at a high rate of 1 A g^−1^, a capacity of 133.3 mAh g^−1^ is maintained (Fig. 3e). It is evident that MnO_*x*_ displays greater stability and reversibility than α-MnO_2_ during charging/discharging. Under different current densities, the electrochemical properties of manganese oxides, such as initial specific capacity, maximum specific capacity, and activation process, are different. These phenomena may be due to the different polarizations of the electrodes at different current densities. As compared with most Mn-based Zn-ion batteries (Table S1), the carbon-coated MnO_*x*_ cathode using Mn^2+^-containing electrolyte delivers competitive energy density. The electrochemical performances of MnO_*x*_-1, MnO_*x*_-3, and MnO are provided in Figs. S3–S5.

### Reaction Mechanism

**Fig. 4:**
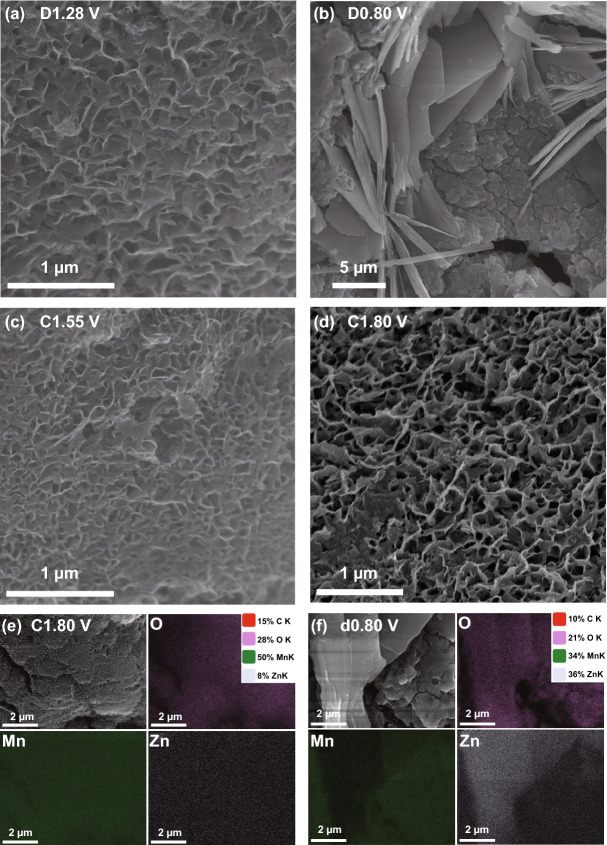
**a**–**d** Ex situ SEM images at different states of MnO_*x*_-2 cathode. EDX elemental (Mn, O, and Zn) mapping images **e** at fully charged state and **f** at fully discharged state

In order to understand the reasons for the superior electrochemical performance of carbon-coated MnO_*x*_ nanoparticles, the ex situ SEM, ex situ XRD, ex situ XPS, and ex situ inductively coupled plasma optical emission spectroscopy (ICP-OES) at different cycling states were conducted to reveal the morphology and crystal structure evolution of the MnO_*x*_ cathode. Figure 4 shows the ex situ SEM images of the MnO_*x*_-2 cathode materials at different cycling stages. As shown in Fig. 4a, the nanosheet array covers the electrode surface when discharging to 1.28 V. But the nanosheet array structure disappears and the electrode surface is covered by new flake-like compounds in the fully discharged stage (Fig. 4b). When charging to 1.55 V (Fig. 4c), the nanosheet arrays are regenerated. And thicker active materials with nanosheet structure are generated on the electrode surface in the fully charged stage (Fig. 4d). The nanosheet-like structure formed in situ during the charge process possesses a high specific surface area, which can facilitate electron transport and shorten the ion diffusion length. The EDX elemental (Mn, Zn, and O) mapping images at different charged/discharge states are shown in Figs. 4e, f, and S7, S8. At the fully charged state, the electrodes are covered with nanosheets, and Mn and O elements are distributed on the nanosheets, but there is almost no Zn element. On the contrary, at the fully discharged state, Zn element is distributed on the flake-like substance, and Mn element is also present on the electrode, which is due to the presence of unoxidized MnO in the electrode.

**Fig. 5:**
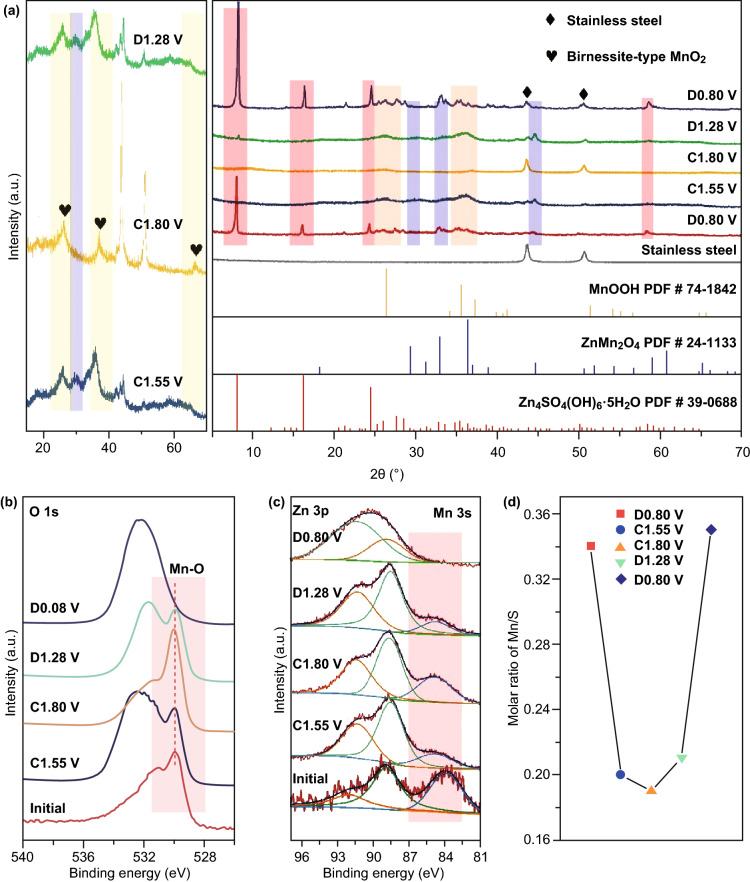
**a** Ex situ XRD patterns of the third cycle at 0.05 A g^−1^ of MnO_*x*_-2 cathode. XPS spectra of **b** O 1*s* and **c** Mn 3*s*/Zn 3*p* under different states of MnO_*x*_-2 cathode. **d** Molar ratios of Mn/S in the electrolytes under different states of MnO_*x*_-2 cathode

Figure 5a displays the ex situ XRD patterns of MnO_*x*_ electrode at different charge and discharge states. First, in the fully discharged stage (0.80 V), the XRD peaks are in good agreement with Zn_4_SO_4_(OH)_6_·5H_2_O (JCPDS No. 39-0688) phase, proving that the flake-like compounds are Zn_4_SO_4_(OH)_6_·5H_2_O. After charging to 1.55 V, phases of ZnMn_2_O_4_ (JCPDS No. 24-1133) and MnOOH (JCPDS No. 74-1842) are observed. But in the fully charged stage (1.80 V), both intermediate phases, ZnMn_2_O_4_ and MnOOH, evolve into low-crystallinity MnO_2_ with birnessite structures [32]. During the subsequence discharge process, ZnMn_2_O_4_ and MnOOH diffraction peaks re-emerge when discharging to 1.28 V, indicating a good reversibility of electrode reaction. Finally, at full depth of discharge, the regeneration of Zn_4_SO_4_(OH)_6_·5H_2_O is seen in the ex situ XRD. Combined with the ex situ SEM results, the ex situ XRD patterns of MnO_*x*_ electrode reveal the reversible Mn^2+^/Mn^4+^ double redox (birnessite-type MnO_2_ ↔ monoclinic MnOOH and spinel ZnMn_2_O_4_ ↔ Mn^2+^ ions).

The ex situ XPS spectra at different states are collected to gain insight into the redox behaviour of MnO_*x*_ electrode. Due to the overlap of Zn 3*p*, it is difficult to consistently resolve the average oxidation state of Mn at different states of charge [33]. However, it is apparent that the peak intensities of both Mn–O bond (Fig. 5b) and Mn 3*s* (Fig. 5c) increase during the charge process, and the tendency reversed during the subsequent discharge process. As shown in Fig. 5d, the molar ratios of Mn/S in the electrolyte at different stages are also analyzed by ICP-OES to strongly demonstrate the reversible Mn^2+^/Mn^4+^ double redox. In the fully discharged stage (0.8 V), the molar ratio of Mn/S is the highest. After charged to 1.55 V, the molar ratio of Mn/S declines precipitously. When charged to the fully charged stage (1.8 V), the molar ratio of Mn/S decreases slightly. As the electrode is discharged to 1.28 V, the molar ratio of Mn/S shows a slight rebound. After fully discharged again, a significant recovery on the molar ratio of Mn/S is observed, and the ratio is slightly higher than that of the last fully discharged state. It further supports that most of the Mn^2+^ ions in the electrolyte are consumed to form the monoclinic MnOOH and spinel ZnMn_2_O_4_ phase due to the electro-oxidation process. During the following charge stages, the redox reactions between the ZnMn_2_O_4_ spinel phase (MnOOH phase) and birnessite phases cause a slight decrease of the ratio. Subsequent recovery corresponded to the dissolution of ZnMn_2_O_4_ phase and MnOOH phase into the electrolyte. Based on the above analysis, it is reasonable to conclude that manganese deposition and dissolution occurred during charge and discharge.

**Fig. 6:**
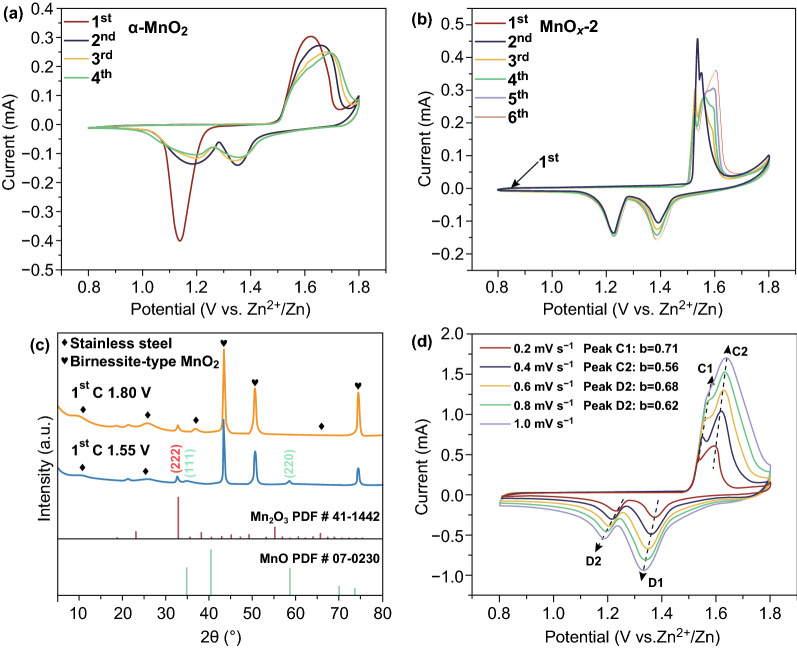
CV curves of **a** α-MnO_2_ electrode at 0.1 mV s^−1^ and **b** MnO_*x*_-2 electrode at 0.1 mV s^−1^. **c** Ex situ XRD patterns of the first cycle. **d** CV curves of the MnO_*x*_-2 cathode at different sweep rates

The cyclic voltammetry (CV) is used to further analyze the difference in electrochemical behavior between α-MnO_2_ and MnO_*x*_-2. For α-MnO_2_ (Fig. 6a), similar to most MnO_2_ cathodes, its open-circuit voltage is 1.36 V. The current response observed at 1.14 V is associated with the formation of monoclinic MnOOH or spinel ZnMn_2_O_4_ in the initial cathodic polarization process [34, 35]. In the initial anodic sweep, the current response observed at 1.62 V is similar to the following three scans for α-MnO_2_ electrode, which is ascribed to the extraction process of H^+^ or Zn^2+^ [36, 37]. The reactions can be formulated as follows:

1 $${\text{MnOOH }} \leftrightarrow {\text{ MnO}}_{2} + {\text{ H}}^{ + } + {\text{ e}}^{ - }$$

2 $${\text{ZnMn}}_{2} {\text{O}}_{4} \leftrightarrow {\text{ Zn}}^{2 + } + \, 2{\text{MnO}}_{2} + \, 2{\text{e}}^{ - }$$

Interestingly, the MnO_*x*_ cathode has a low open-circuit voltage of 0.88 V. The currents are very strong at 1.53 and 1.55 V in the initial anodic sweep (Fig. 6b), which are related to the consequent oxidations of Mn^2+^ to Mn^3+^ and Mn^4+^. The XRD patterns of MnO_*x*_ electrode during the first charge process are shown in Fig. 6c. The patterns demonstrate the emerge of low-crystallinity birnessite-type MnO_2_. And we propose the following possible reaction pathways:

3 $$3 {\text{MnO }} \to {\text{ Mn}}_{ 2} {\text{O}}_{ 3} + {\text{ Mn}}^{ 2+ } + {\text{ 2e}}^{ - }$$

4 $$2 {\text{Mn}}_{ 2} {\text{O}}_{ 3} \to {\text{ 3MnO}}_{ 2} + {\text{ Mn}}^{ 2+ } + {\text{ 2e}}^{ - }$$

Combined with the ex situ XRD results, the two well-defined cathodic peaks at 1.23 and 1.38 V and anodic peaks near 1.52 and 1.60 V correspond to the two-step electrochemical reaction between Mn^2+^ and Mn^4+^. Based on the above discussions, the energy storage mechanism of MnO_*x*_ electrode is described as follows:

5 $$2 {\text{MnO}}_{ 2} + {\text{ Zn}}^{ 2+ } + {\text{ 2e}}^{ - } \leftrightarrow {\text{ ZnMn}}_{ 2} {\text{O}}_{ 4}$$

6 $${\text{MnO}}_{ 2} + {\text{ H}}^{ + } + {\text{ e}}^{ - } \leftrightarrow {\text{ MnOOH}}$$

7 $$3 {\text{ZnMn}}_{ 2} {\text{O}}_{ 4} + {\text{ 4SO}}_{ 4}^{ 2- } + {\text{ 32H}}_{ 2} {\text{O }} + {\text{ 13Zn}}^{ 2+ } + {\text{ 6e}}^{ - } \leftrightarrow {\text{ 6Mn}}^{ 2+ } + {\text{ 4Zn}}_{ 4} {\text{SO}}_{ 4} \left( {\text{OH}} \right)_{ 6} \cdot 5 {\text{H}}_{ 2} {\text{O}}$$

8 $$2 {\text{MnOOH }} + {\text{ SO}}_{ 4}^{ 2- } + {\text{ 7H}}_{ 2} {\text{O }} + {\text{ 4Zn}}^{ 2+ } + {\text{ 2e}}^{ - } \leftrightarrow {\text{ 2Mn}}^{ 2+ } + {\text{ Zn}}_{ 4} {\text{SO}}_{ 4} \left( {\text{OH}} \right)_{ 6} \cdot 5 {\text{H}}_{ 2} {\text{O}}$$

Apparently, stronger peak intensity is observed in MnO_*x*_-2 electrode, indicating its higher electrochemical reactivity and higher capacity [38]. In addition, the overpotential gaps of MnO_*x*_-2 electrode are smaller than that of α-MnO_2_ electrode. The higher reactivity and smaller polarization of MnO_*x*_-2 may be caused by the low crystallinity of in situ generated birnessite-type MnO_2_.

As shown in Fig. 6d, the CV curves of the MnO_*x*_ at different scanning rates are further used to determine the electrochemical behavior. In general, the peak current (*i*) obeys an empirical power-law relationship with the scan rate (*v*):

9 $$i \, = \, av^{b}$$

10 $$\log \left( i \right) \, = \, b \, \log \left( v \right) \, + \, \log \left( a \right)$$

The parameter *b* determined by the plots of log (*i*) and log (*ν*) reflects the dominated diffusion modes [39, 40]. And the parameter *b* for both anodic and cathodic peaks is calculated to be 0.71, 0.56, 0.68, and 0.62, respectively. The *b*-value of the four peaks is close to 0.5, demonstrating that the conversion reaction and the insertion/extraction of H^+^ and Zn^2+^ are controlled by diffusion.

## Conclusions

In summary, a rechargeable aqueous zinc–manganese battery with promising electrochemical performance is developed. The low-crystallinity birnessite-type MnO_2_ generated in situ from carbon-coated MnO_*x*_ nanoparticles achieves the reversible Mn^2+^/Mn^4+^ double redox. The mechanism involves a reversible double redox between Mn^2+^ and birnessite-type MnO_2_. Benefitting from the reversible Mn^2+^/Mn^4+^ double redox, the MnO_*x*_ cathode using Mn^2+^-containing ZnSO_4_ electrolyte exhibits excellent electrochemical properties with superior cycling stability and high capacity in comparison with most of the reported cathodes for AZIBs. The analysis of electrochemical reaction mechanism will open a promising avenue to further enhance the energy density of aqueous batteries. The overall combination of low-cost MnO_*x*_ cathode materials, mild aqueous electrolytes, metal Zn anode, and simpler assembly parameters can allow aqueous zinc–manganese batteries meet the requirements of large-scale storage applications.

## Electronic supplementary material

Below is the link to the electronic supplementary material.

Supplementary material 1 (PDF 658 kb)
