# Supplementary material for: High-Performance Aqueous Zinc–Manganese Battery with Reversible Mn2+/Mn4+ Double Redox Achieved by Carbon Coated MnOx Nanoparticles
Source: Nanomicro Lett. 2020 May 13;12:110. doi: 10.1007/s40820-020-00445-x (PMC7770770; doi:10.1007/s40820-020-00445-x)
Supplement: Supplementary file 1 — Supplementary material 1 (PDF 658 kb) [file 40820_2020_445_MOESM1_ESM.pdf]

Supporting Information for

High-Performance Aqueous Zinc-Manganese Battery with Reversible Mn^{2+}/Mn^{4+} Double Redox Achieved by Carbon Coated MnO_x Nanoparticles

Jingdong Huang[1, a], Jing Zeng[1, a], Kunjie Zhu[2], Ruizhi Zhang[3, *], Jun Liu[1, *]

[1]School of Materials Science and Engineering, Central South University, Changsha 410083, People‘s Republic of China

[2]Key Laboratory of Advanced Energy Materials Chemistry (Ministry of Education), College of Chemistry, Nankai University, Tianjin 300071, People‘s Republic of China

[3]Hunan institute of technology, Hengyang 421002, People‘s Republic of China

[a]Jingdong Huang and Jing Zeng contributed equally to this work

[*] Corresponding authors.E-mail: zhangruizhi@gmail.com (Ruizhi Zhang); liujun4982004@csu.edu.cn (Jun Liu)

Supplementary Figures

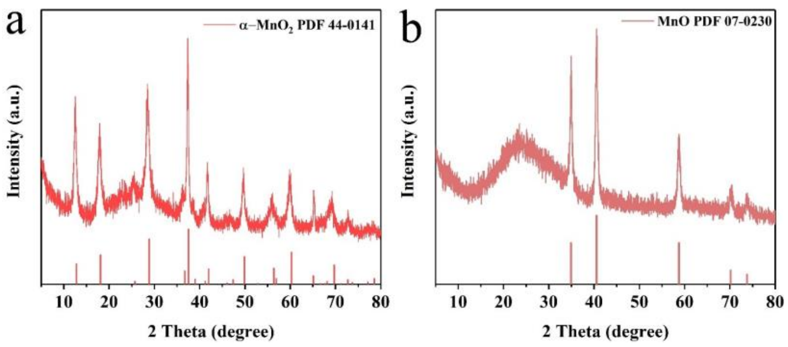

Fig. S1 XRD patterns of **a** α-MnO_2 and **b** MnO

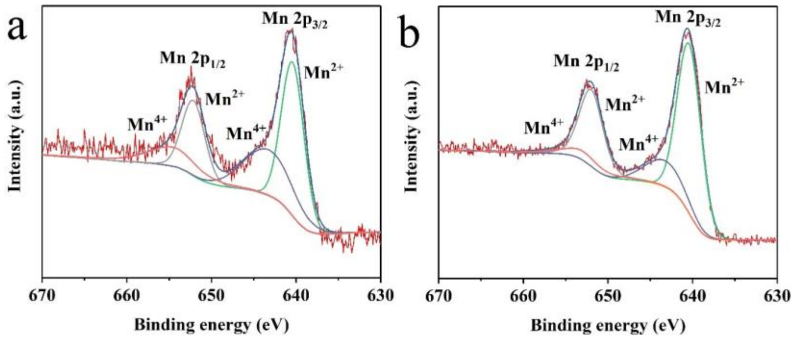

Fig. S2 XPS spectra showing the 2*p* core-level spectra of Mn in **a** MnO_x-1 and **b** MnO_x-3

Table S1 Comparison for electrochemical performances of representative Mn-O cathode materials in Zinc ion battery and our present work

Cathode material	Electrolytes	Capacity	Max Energy density	Max Power density	Capacity retention
α-MnO_2 [S1]	1 M $ZnSO_4$	353 mAh g^{-1} at 16 mA g^{-1}	—	—	63% after 50 cycles
α- MnO_2 [S2]	0.1 M $Zn(NO_3)_2$	210 mAh g^{-1} at 0.5C	—	—	100% after 100 cycles
α- MnO_2 [S3]	1 M $ZnSO_4$	323 mAh g^{-1} at 16 mA g^{-1}	—	—	46% after 75 cycles
α- MnO_2 [S4]	2 M $ZnSO_4$ + 0.1 M $MnSO_4$	285 mAh g^{-1} at C/3	~170 Wh kg^{-1} (cathode, anode and electrolyte)	—	92% after 5000 cycles.
β- MnO_2 [S5]	3 M $Zn(CF_3SO_3)_2$ + 0.1 M $Mn(CF_3S_3)_2$	258 mAh g^{-1} at 0.65 C	254 Wh kg^{-1} (based on cathode)	5.9 kW kg^{-1} (based on cathode)	94% after 2000 cycles.
γ- MnO_2 [S6]	1 M $ZnSO_4$	285 mAh g^{-1} at 0.05 mA cm^{-2}	—	—	63% after 45 cycles
ε- MnO_2 [S7]	2 M $ZnSO_4$ + 0.2 M $MnSO_4$	290 mAh g^{-1} at 90 mA g^{-1}	—	—	99.3% after 10000 cycles.
δ- MnO_2 [S8]	1 M $ZnSO_4$	252 mAh g^{-1} at 83 mA g^{-1}	—	—	～44% after 100 cycles
Graphene/α-MnO_2 [S9]	2 M $ZnSO_4$ + 0.2 M $MnSO_4$	382 mAh g^{-1} at 300 mA g^{-1}	406.6 Wh kg^{-1}(based on cathode)	9.5 kW kg^{-1} (based on cathode)	94% after 3000 cycles
MnO_2/PEDOT [S10]	PVA+3 M LiCl+2 M $ZnCl_2$ + 0.4 M $MnSO_4$	367 mA h g^{-1} at 0.74 A g^{-1}	505 Wh kg^{-1} (based on cathode)	8.6 kW kg^{-1} (based on cathode)	83.7% after 300 cycles
Polyaniline-intercalated MnO_2 [S11]	2 M $ZnSO_4$ + 0.1 M $MnSO_4$	280 mA h g^{-1} at 200 mA g^{-1}	—	—	100% after 200 cycles
O_d- MnO_2 [S12]	1 M $ZnSO_4$ + 0.2 M $MnSO_4$	345 mAh g^{-1} at 200 mA g^{-1}	470 Wh kg^{-1} (based on cathode)	10 kW kg^{-1} (based on cathode)	84% after 2000 cycles
Mn_2O_3 [S13]	2 M $ZnSO_4$ + 0.1 M $MnSO_4$	148 mAh g^{-1} at 100 mA g^{-1}	—	—	～68% after 2000 cycles
Mn_3O_4 [S14]	2 M $ZnSO_4$	239 mAh g^{-1} at 100 mA g^{-1}	—	—	—
MnO_x@N-C [S15]	2 M $ZnSO_4$ + 0.1 M $MnSO_4$	385 mAh g^{-1} at 100 mA g^{-1}	—	—	100% after 1600 cycles
D-β-MnO_2 [S16]	3 M $ZnSO_4$ + 0.1 M $MnSO_4$	276 mAh g^{-1} at 100 mA g^{-1}	—	—	94 % after 300 cycles
MnO_2 [S17]	6 M KOH + 0.2 M ZnO + 5 mM vanillin and 3 M H_2SO_4 + 0.1	616 mAh g^{-1} at 100 mA g^{-1}	1,621.7 Wh $kg^{-1}{}_{MnO_2}$	—	96 % after 200 cycles

	M $MnSO_4$)				
G-MnO_2 [S18]	2 M $ZnSO_4$ + 0.1 M $MnSO_4$	321 mAh g^{-1} at 240 mA g^{-1}	—	—	91 % after 300 cycles
P-MnO_{2-x}@VMG [S19]	2 M $ZnSO_4$ + 0.2 M $MnSO_4$	302.8 mAh g^{-1} at 500 mA g^{-1}	—	—	90 % after 1000 cycles
Mn_2O_3 [S20]	2 M $ZnSO_4$ + 0.2 M $MnSO_4$	233 mAh g^{-1} at 300 mA g^{-1}	—	—	89 % after 3000 cycles
Birnessite MnO_2 [S21]	2 M $ZnSO_4$ + 0.5 M $MnSO_4$	279.7 mAh g^{-1} at 300 mA g^{-1}	—	—	61 % after 1500 cycles
Ca_2MnO_4 [S22]	2 M $ZnSO_4$ + 0.1 M $MnSO_4$	250 mAh g^{-1} at 100 mA g^{-1}	—	—	80 % after 1000 cycles
N-MnO_{2-x} [S23]	2 M $ZnSO_4$ + 0.2 M $MnSO_4$	285 mAh g^{-1} at 200 mA g^{-1}	—	—	85.7 % after 1000 cycles
$MnO_2H_{0.16}(H_2O)_{0.27}$ [S24]	1 M $ZnSO_4$ + 0.2 M $MnSO_4$	275.6 mAh g^{-1} at 30.8 mA g^{-1}	228.5 Wh kg^{-1}	—	96 % after 500 cycles
MnO_x (our work)	1 M $ZnSO_4$ + 0.3 M $MnSO_4$	842.5 mAh g^{-1} at 200 mA g^{-1}	1158 Wh kg^{-1} (based on initial cathode)	1.2 kW kg^{-1} (based on initial cathode)	80% after 1500 cycles

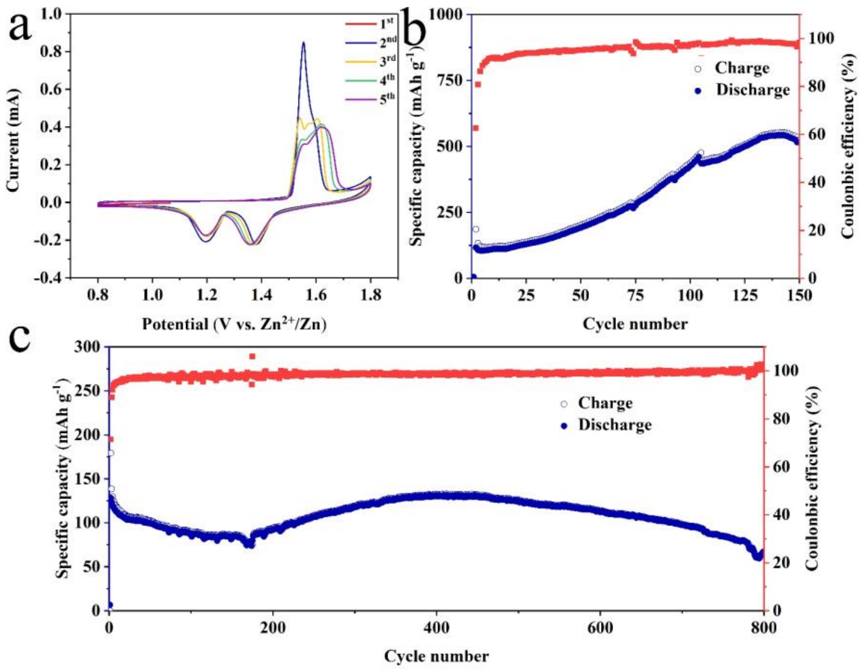

Fig. S3 a CV curses at 0.1 mV s^{-1} in the voltage range of 0.8-1.8 V vs. Zn^{2+}/Zn, **b** cycling performance at 0.2 A g^{-1}; **c** Cycling performance at 1 A g^{-1} of MnO_x-1

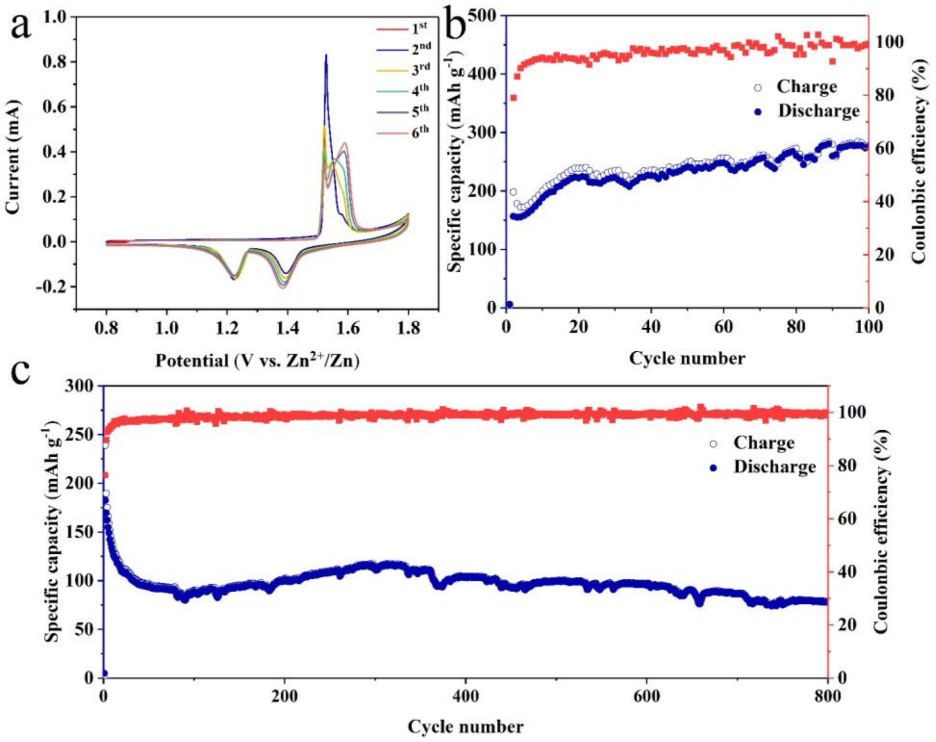

Fig. S4 a CV curses at 0.1 mV s^{-1} in the voltage range of 0.8-1.8 V vs. Zn^{2+}/Zn, **b** cycling performance at 0.2 A g^{-1}; **c** Cycling performance at 0.5 A g^{-1} of MnO_x-3

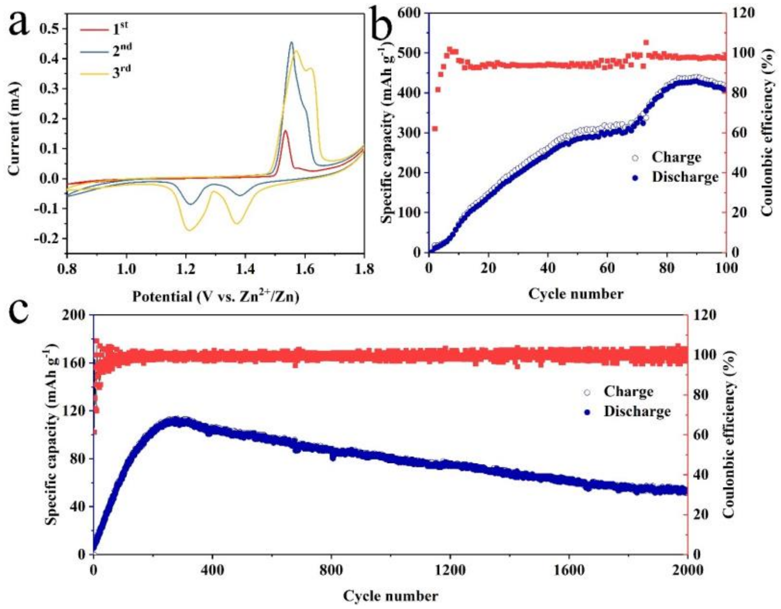

Fig. S5 a CV curses at 0.1 mV s^{-1} in the voltage range of 0.8-1.8 V vs. Zn^{2+}/Zn, **b** cycling performance at 0.2 A g^{-1}; **c** Cycling performance at 1 A g^{-1} of MnO

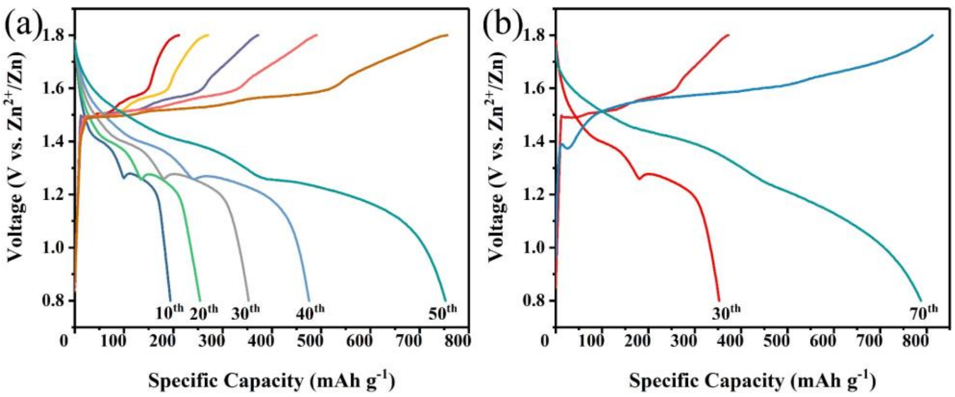

Fig. S6 Voltage profile of MnO_x-2

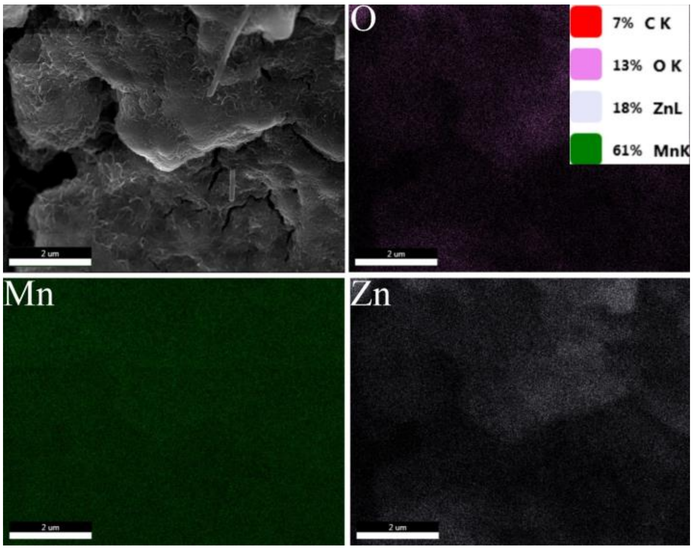

Fig. S7 *ex*-situ SEM image and EDX elemental mapping images of MnO_x-2 when discharging to 1.28 V

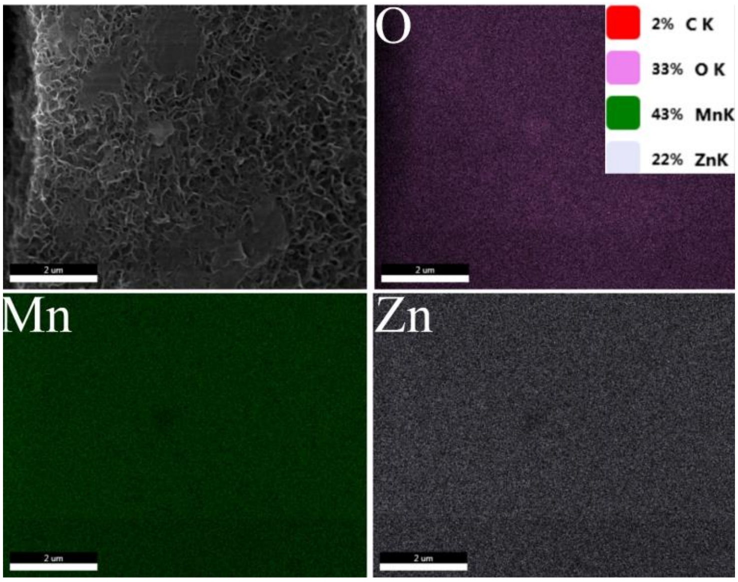

Fig. S8 *ex*-situ SEM image and EDX elemental mapping images of MnO_x-2 when charging to 1.55 V

Supplementary References

[S1] M.H. Alfaruqi, J. Gim, S.Kim, J. Song, J. Jo, S. Kim, V. Mathew, J. Kim, Enhanced reversible divalent zinc storage in a structurally stable α-MnO_2 nanorod electrode. J. Power Sources **288,** 320–327 (2015). https://doi.org/10.1016/j.jpowsour.2015.04.140

[S2] C. Xu, B. Li, H. Du, F. Kang, Energetic zinc ion chemistry: the rechargeable zinc ion battery. Angew. Chem. Int. Ed. **51,** 933–935 (2012). https://doi.org/10.1002/anie.201106307

[S3] M.H. Alfaruqi, S. Islam, J. Gim, J. Song, S. Kim, D.T. Pham, J. Jo, Z. Xiu, V. Mathew, J. Kim, A high surface area tunnel-type α-MnO_2 nanorod cathode by a simple solvent-free synthesis for rechargeable aqueous zinc-ion batteries. Chem. Phys. Lett. **650,** 64–68 (2016). https://doi.org/10.1016/j.cplett.2016.02.067

[S4] H. Pan, Y. Shao, P. Yan, Y. Cheng, K. Han et al., Reversible aqueous zinc/manganese oxide energy storage from conversion reactions. Nat. Energy **1,** 16039 (2016). https://doi.org/10.1038/nenergy.2016.39

[S5] N. Zhang, F. Cheng, J. Liu, L. Wang, X. Long, X. Liu, F. Li, J. Chen, Rechargeable aqueous zinc-manganese dioxide batteries with high energy and power densities. Nat. Commun. **8,** 405 (2017). https://doi.org/10.1038/s41467-017-00467-x

[S6] M.H. Alfaruqi, V. Mathew, J. Gim, S Kim, J Song, J. Baboo, S. Choi, J Kim, Electrochemically induced structural transformation in a γ-MnO_2 cathode of a high capacity zinc-ion battery system. Chem. Mater. **27,** 3609–3620 (2015). https://doi.org/10.1021/cm504717p

[S7] W. Sun, F. Wang, S. Hou, C. Yang, X. Fan et al., Zn/MnO_2 battery chemistry with H^+ and Zn^{2+} coinsertion. J. Am. Chem. Soc. **139,** 9775–9778 (2017). https://doi.org/10.1021/jacs.7b04471

[S8] M.H. Alfaruqi, J. Gim, S. Kim, J. Song, D.T. Pham et al., A layered δ-MnO_2 nanoflake cathode with high zinc-storage capacities for eco-friendly battery applications. Electrochem. Commun. **60,** 121–125 (2015). https://doi.org/10.1016/j.elecom.2015.08.019

[S9] B. Wu, G. Zhang, M. Yan, T. Xiong, P. He, L. He, X. Xu, L. Mai, Graphene scroll-coated α-MnO_2 nanowires as high-performance cathode materials for aqueous Zn-ion battery. Small **14,** 1703850 (2018). https://doi.org/10.1002/smll.201703850

[S10] Y. Zeng, X. Zhang, Y. Meng, M. Yu, J. Yi, Y. Wu, X. Lu, Y. Tong, Achieving ultrahigh energy density and long durability in a flexible rechargeable quasi-solid-state Zn-MnO_2 battery. Adv. Mater. **29**, 1700274 (2017).

https://doi.org/10.1002/adma.201700274

[S11] J. Huang, Z. Wang, M. Hou, X. Dong, Y. Liu, Y. Wang, Y. Xia, Polyaniline-intercalated manganese dioxide nanolayers as a high-performance cathode material for an aqueous zinc-ion battery. Nat. Commun. **9,** 2906 (2018). https://doi.org/10.1038/s41467-018-04949-4

[S12] T. Xiong, Z. Yu, H. Wu, Y. Du, Q. Xie et al., Defect engineering of oxygen-deficient manganese oxide to achieve high-performing aqueous zinc ion battery. Adv. Energy Mater. **9,** 1803815 (2019). https://doi.org/10.1002/aenm.201803815

[S13] B. Jianga, C. Xua, C. Wua, L. Dong, J. Lia, F. Kang, Manganese sesquioxide as cathode material for multivalent zinc ion battery with high capacity and long cycle life. Electrochim. Acta **229,** 422–428 (2017). https://doi.org/10.1016/j.electacta.2017.01.163

[S14] J. Hao, J. Mou, J. Zhang, L. Dong, W. Liu, C. Xu, F. Kang, Electrochemically induced spinel-layered phase transition of Mn_3O_4 in high performance neutral aqueous rechargeable zinc battery. Electrochim. Acta **259,** 170–178 (2018). https://doi.org/10.1016/j.electacta.2017.10.166

[S15] Y. Fu, Q. Wei, G. Zhang, X. Wang, J. Zhang et al., High-performance reversible aqueous Zn-ion battery based on porous MnO_x nanorods coated by MOF-derived N-doped carbon. Adv. Energy Mater. **8,** 1801445 (2018). https://doi.org/10.1002/aenm.201801445

[S16] M. Han, J. Huang, S. Liang, L Shan, X. Xie, Z. Yi, Y. Wang, S. Guo, J. Zhou, Oxygen defects in β-MnO_2 enabling high-performance rechargeable aqueous zinc/manganese dioxide battery. Iscience **23,** 100797 (2020). https://doi.org/10.1016/j.isci.2019.100797

[S17] C. Zhong, B. Liu, J. Ding, X. Liu, Y. Zhong et al., Decoupling electrolytes towards stable and high-energy rechargeable aqueous zinc–manganese dioxide batteries. Nat. Energy 1-10 (2020). https://doi.org/10.1038/s41560-020-0584-y

[S18] C. Wang, M. Wang, Z. He, L. Liu, Y. Huang, Rechargeable aqueous zinc-manganese dioxide/graphene batteries with high rate capability and large capacity. ACS Appl. Energy Mater. **3,** 1742-1748 (2020). https://doi.org/10.1021/acsaem.9b02220

[S19] Y. Zhang, S. Deng, G. Pan, H. Zhang, B. Liu et al., Introducing oxygen defects into phosphate ions intercalated manganese dioxide/vertical multilayer graphene arrays to boost flexible zinc ion storage. Small 1900828 (2020). https://doi.org/10.1002/smtd.201900828

[S20] D. Feng, T. Gao, L. Zhang, B. Guo, S. Song, Z. Qiao, S. Dai, Boosting high-rate zinc-storage performance by the rational design of Mn_2O_3

nanoporous architecture cathode. Nano-Micro Lett. **12,** 14 (2020). https://doi.org/10.1007/s40820-019-0351-4

[S21] G. Li, Z. Huang, J. Chen, F. Yao, J. Liu, O. Li, S. Sun, Z. Shi, Rechargeable Zn-ion batteries with high power and energy density: a two-electron reaction pathway in birnessite MnO_2 cathode materials. J. Mater. Chem. A **8,** 1975-1985 (2020). https://doi.org/10.1039/C9TA11985J

[S22] S. Guo, S. Liang, B. Zhang, G. Fang, D. Ma, J. Zhou, Cathode interfacial layer formation via in situ electrochemically charging in aqueous zinc-ion battery. ACS Nano **13,** 13456-13464 (2019). https://doi.org/10.1021/acsnano.9b07042

[S23] Y. Zhang, S. Deng, M. Luo, G. Pan, Y. Zeng et al., Defect promoted capacity and durability of N-MnO_{2-x} branch arrays via low-temperature NH_3 treatment for advanced aqueous zinc ion batteries. Small **15,** 1905452 (2019). https://doi.org/10.1002/smll.201905452

[S24] Q. Zhao, X. Chen, Z. Wang, L. Yang, R. Qin et al., Unravelling H^+/Zn^{2+} synergistic intercalation in a novel phase of manganese oxide for high-performance aqueous rechargeable battery. Small **15,** 1904545 (2019). https://doi.org/10.1002/smll.201904545
